# Extracellular IL-33 cytokine, but not endogenous nuclear IL-33, regulates protein expression in endothelial cells

**DOI:** 10.1038/srep34255

**Published:** 2016-10-03

**Authors:** Violette Gautier, Corinne Cayrol, Dorian Farache, Stéphane Roga, Bernard Monsarrat, Odile Burlet-Schiltz, Anne Gonzalez de Peredo, Jean-Philippe Girard

**Affiliations:** 1 Institut de Pharmacologie et de Biologie Structurale, Université de Toulouse, CNRS, UPS, Toulouse, France

## Abstract

IL-33 is a nuclear cytokine from the IL-1 family that plays important roles in health and disease. Extracellular IL-33 activates a growing number of target cells, including group 2 innate lymphoid cells, mast cells and regulatory T cells, but it remains unclear whether intracellular nuclear IL-33 has additional functions in the nucleus. Here, we used a global proteomic approach based on high-resolution mass spectrometry to compare the extracellular and intracellular roles of IL-33 in primary human endothelial cells, a major source of IL-33 protein in human tissues. We found that exogenous extracellular IL-33 cytokine induced expression of a distinct set of proteins associated with inflammatory responses in endothelial cells. In contrast, knockdown of endogenous nuclear IL-33 expression using two independent RNA silencing strategies had no reproducible effect on the endothelial cell proteome. These results suggest that IL-33 acts as a cytokine but not as a nuclear factor regulating gene expression in endothelial cells.

Interleukin-33 (IL-33) is a tissue-derived nuclear cytokine from the IL-1 family with critical roles in tissue homeostasis and repair, type 2 immunity, viral infection, inflammation and allergy^1^,^2^,^3^,^4^,^5^. IL-33 binds to the ST2 receptor expressed on cells of the innate and adaptive immune system^1^. Tissue-resident cells such as group 2 innate lymphoid cells (ILC2s), mast cells, and certain subsets of regulatory T cells, constitutively express high levels of ST2 and are major targets of IL-33 *in vivo*^6^,^7^,^8^,^9^,^10^,^11^,^12^,^13^,^14^. Other targets of IL-33 include macrophages, dendritic cells, Th2 cells, eosinophils, basophils, NK and iNKT cells, neutrophils, Th1 cells and CD8^+^ T cells^4^. Studies in humans and animal models suggest a critical role of IL-33 in many important diseases^3^,^4^,^12^,^14^,^15^,^16^,^17^,^18^,^19^,^20^. The genes encoding *IL-33* and *ST2* have been reproducibly identified as major susceptibility loci for human asthma in several genome-wide association studies^3^,^15^. IL-33 also appears to be important for other allergic diseases (allergic rhinitis, atopic dermatitis, allergic conjunctivitis), adipose tissue metabolism and obesity, and a variety of diseases associated with tissue injury and repair (myocardial infarction, stroke, wounding, microbial infection, hepatic and pulmonary fibrosis, systemic sclerosis, chronic obstructive pulmonary disease, autoimmune diseases and cancer)^3^,^4^,^12^,^16^,^17^,^18^,^19^,^20^. Given these critical roles in health and disease, a good understanding of IL-33 biology and mode of action is crucial.

IL-33 is constitutively expressed in the nuclei of producing cells during homeostasis, including epithelial cells from various barrier tissues, endothelial cells from blood vessels, fibroblastic reticular cells of lymphoid organs, and post-mitotic oligodendrocytes in the brain^21^,^22^,^23^,^24^. Although already high during homeostasis, expression of IL-33 is further upregulated during inflammation, and the protein can be produced by additional cell types^3^,^4^,^23^,^25^. Full length IL-33 is biologically active and it can be released from the nucleus of producing cells after cellular damage or necrotic cell death^26^,^27^. It was thus proposed to function as an alarm signal (alarmin) that alerts immune cells of tissue damage^21^,^26^,^27^. IL-33 cytokine activity is regulated by nuclear compartmentalization or sequestration^28^ and proteolytic maturation^3^. During apoptosis, IL-33 is inactivated by caspases that cleave the protein within the IL-1-like cytokine domain^26^,^27^. During inflammation, IL-33 is processed in the central activation domain by inflammatory proteases from mast cells and neutrophils, that generate mature forms of the protein with 10 to 30 fold higher biological activity^29^,^30^. Moreover, mature forms of IL-33 are rapidly inactivated (<2 h) in the extracellular environment by oxidation of critical cysteine residues^31^.

Nuclear localization of IL-33 is a fundamental property of the protein that has been observed in all producing cells, both in human and mouse tissues^21^,^22^,^23^. In the nucleus, IL-33 associates with chromatin through a short chromatin-binding motif that recognizes the heterodimer formed by histones H2A and H2B^32^. Evolutionary conservation of the N-terminal nuclear domain of IL-33, that contains the chromatin-binding motif MXLRSG, strictly conserved in all IL-33 sequences^2^, suggests a critical role for nuclear localization and chromatin association. Previously, we demonstrated that nuclear IL-33 exhibits transcriptional repressor properties when overexpressed in transfected HEK293 cells^2^, a finding confirmed by others^33^. We thus proposed that IL-33 may be a dual function protein, acting both extracellularly as an IL-1 family cytokine, and intracellularly as a nuclear factor regulating gene expression^2^. Nuclear functions of IL-33 in transcriptional regulation have been proposed in several recent studies^34^,^35^,^36^. However, to date, no large scale study has been performed to demonstrate a global role of endogenous nuclear IL-33 in the regulation of gene or protein expression.

Here, we used a high-throughput proteomic approach to address this important question. We selected human primary endothelial cells for these studies because they express endogenous nuclear IL-33 constitutively^22^,^26^ and can also respond to extracellular IL-33 cytokine^37^,^38^,^39^. We could thus compare the activities of extracellular and intracellular IL-33 in a unique cellular system and using the same global approach. We found that extracellular IL-33 induced expression of many inflammatory proteins in stimulated endothelial cells. In contrast, knockdown of endogenous nuclear IL-33 using two independent RNA silencing strategies did not induce reproducible changes in the endothelial cell proteome. Together, these proteome-wide analyses do not support the previous proposal that IL-33 is a dual function protein. They suggest that the main purpose of IL-33 nuclear localization is the regulation of its extracellular cytokine activity through nuclear sequestration, rather than the regulation of gene or protein expression.

## Results

### A global proteomic approach to analyze IL-33 function in endothelial cells

**Figure 1:**
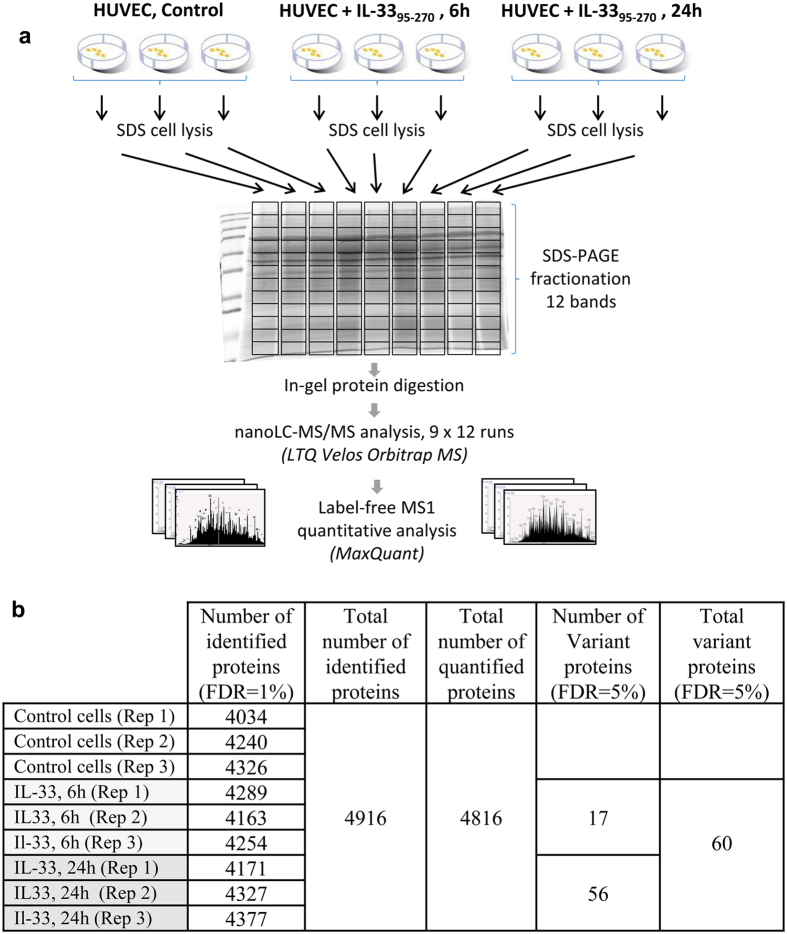
High throughput mass spectrometry analysis of the endothelial cell proteome after stimulation with extracellular IL-33 cytokine. (**a**) Experimental design. Three independent biological experiments were performed by stimulating primary human endothelial cells (HUVECs) with 100 ng/ml IL-33 mature form IL-33_95–270_ during 6 h or 24 h. Total cell lysates from control and stimulated samples were loaded and fractionated on nine parallel gel lanes, cut into 12 bands. The 12 gel bands were digested, and each of the corresponding peptide digests was analyzed by nanoLC-MS/MS. (**b**) Identification results of the large-scale quantitative proteomic analyses. The table indicates the number of proteins identified for each condition, and the total number of proteins identified and quantified in each experiment, as well as the number of variant proteins (proteins detected as differentially expressed with a FDR adjusted at 5%).

**Figure 2:**
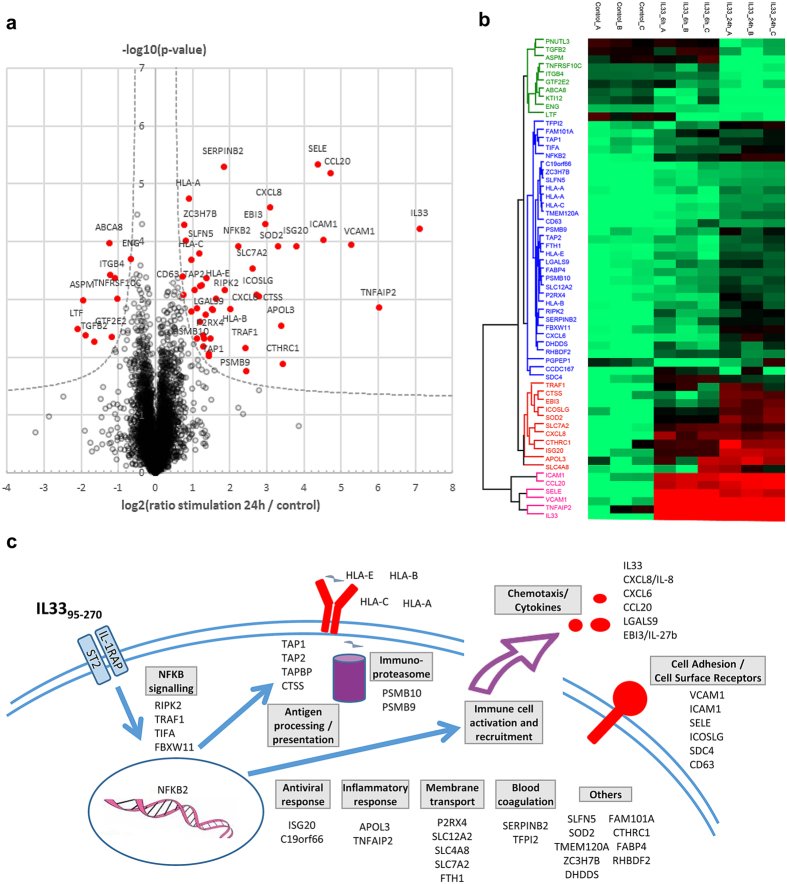
Proteomic analysis of the endothelial cell response to the stimulation with extracellular IL-33 mature form IL-33_95–270_. (**a**) Volcano plot (-log10(p-value) versus log2(fold change)) showing protein expression changes in primary human endothelial cells stimulated for 24 h with 100 ng/ml IL-33_95–270_ compared to non-stimulated cells. Statistical analysis was performed from 3 biological replicates by two-sided Student t-test, variance correction and permutation-based FDR control in Perseus. Proteins considered as significantly regulated (FDR<5%, hyperbolic selection curve indicated in grey) are plotted in red. (**b**) Heat-map of protein intensity following hierarchical clustering of significantly regulated proteins. For each protein the log_2_(intensity) measured in the 3 biological replicates (noted A, B, C) is shown for control cells (non-stimulated), and for cells stimulated 6 h or 24 h. Log2 (intensity) values were scaled before clustering by adjusting the lower bound of the 9 data points to zero. Clusters shown on the left illustrate down- regulated proteins (green), moderately up-regulated proteins with maximal induction at 24 h (blue) and strongly up-regulated proteins with early induction (bottom clusters). (**c**) Schematic illustration of the IL33-induced inflammatory response in primary human endothelial cells. All proteins found to be significantly up-regulated after treatment with IL-33_95–270_ for 6 h or 24 h are represented.

We used a large scale label-free proteomic approach^40^ to analyze the effect of extracellular IL-33 cytokine and endogenous nuclear IL-33 on the endothelial cell proteome. In a first series of experiments (Fig. 1a), we stimulated primary human endothelial cells with 100 ng/ml IL-33 mature form IL-33_95–270_^29^,^30^ during 6 h (IL-33-6h) or 24 h (IL-33-24h). IL-33 mature form IL-33_95–270_^29^,^30^ was used for these experiments because it is a natural form of human IL-33 that can be generated by both neutrophil and mast cell proteases^29^,^30^ (as opposed to the artificially truncated form IL-33_112–270_^1^ that may not exist *in vivo*). Cells cultured in the absence of IL-33 were used as control. Samples were analyzed by nanoLC-MS/MS on Orbitrap-Velos high speed mass spectrometers, and three biological replicates (independent biological stimulations using the same donors) were performed to increase the robustness of our MS-based peptide quantification method^41^. Good technical and biological repeatability could be obtained between the replicate experiments (Supplementary Fig. 1). We could identify up to 4916 distinct proteins in control or IL-33-stimulated endothelial cells (Fig. 1b). Quantitative analysis was performed with the MaxQuant software through the MaxLFQ algorithm that uses optimized procedures for integration and normalization of mass spectrometry data obtained from fractionated samples^42^. The resulting protein intensity values (LFQ metric) were used for quantification, and only proteins repeatedly quantified in the three replicates of at least one experimental condition (Control, IL-33-6h, or IL-33-24h) were used for further statistical analysis. For defining expression changes in pairwise comparisons, a Student t-test was calculated in the Perseus software and a permutation-based approach was used to account for multiple testing and adjust the final false discovery rate at 5%. Based on this stringent selection procedure, 56 proteins were reproducibly found to exhibit a significant variation in endothelial cells stimulated with IL-33_95–270_ during 24 h (Fig. 2a,b and Supplementary Table 1). 17 proteins were already modulated after 6 h of stimulation with IL-33_95–270_ (Supplementary Fig. 2). Interestingly, many proteins modulated by IL-33 in primary human endothelial cells (Fig. 2c) are also regulated by IL-1β or a combination of TNFα and INFγ^40^, indicating that extracellular IL-33 acts principally as a pro-inflammatory cytokine in endothelial cells (see below). We concluded that the high-throughput proteomic approach that we have developed (this study, ref. 40) is an appropriate strategy to identify IL-33 effects on protein expression on a global scale.

### Extracellular IL-33 cytokine induces expression of inflammatory proteins in endothelial cells

Analysis of the proteomic data indicated that extracellular IL-33 induces expression of many proteins associated with inflammatory responses in endothelial cells (Fig. 2a–c). These included cell adhesion receptors involved in leukocyte/endothelium interactions during inflammation (SELE/E-selectin, ICAM1, VCAM1, ICOS ligand), chemokines and cytokines (CXCL6, CCL20, CXCL8/IL-8, EBI3/IL-27beta, Galectin-9), MHC class I molecules (HLA-A, HLA-B, HLA-C, HLA-E) and proteins involved in antigen processing and presentation (immunoproteasome subunits PSMB9 and PSMB10, peptide transporters TAP1 and TAP2, Tapasin TABP). In addition, one NFkB subunit (NFkB2) and several proteins involved in signaling pathways leading to activation of the NFkB pathway (RIPK2, TRAF1, TIFA, FBXW11) were also upregulated after exposure to IL-33. As a matter of fact, many of the induced proteins that we identified (EBI3/IL-27beta, SELE, VCAM1, ICAM1, TNFAIP2, SOD2, TRAF1, HLA-B, SDC4, RIPK2, TABP, TAP1, IL-8) are known targets of NFkB (Fig. 2c). Therefore, our proteome-wide analyses showed that the NFkB pathway is the major cellular pathway activated by extracellular IL-33 cytokine in endothelial cells.

**Figure 3:**
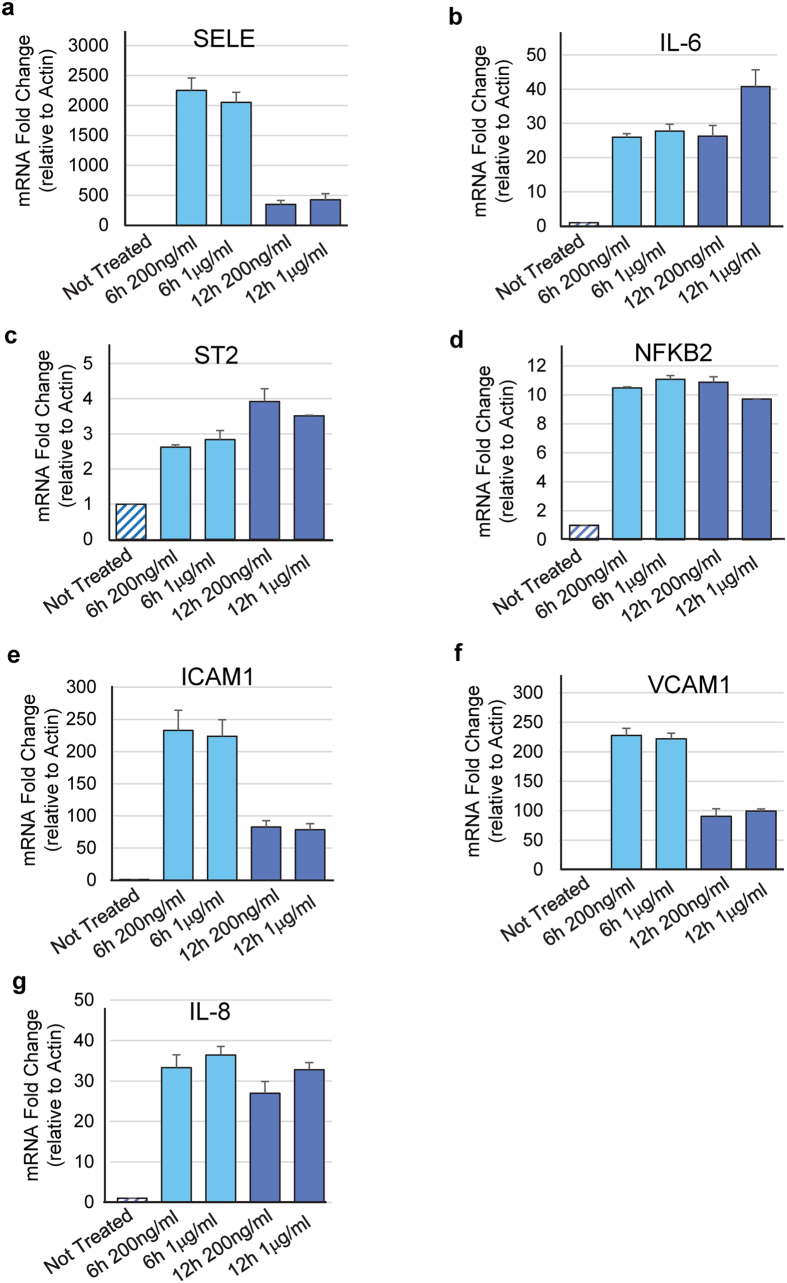
Relative mRNA expression of different genes regulated by extracellular IL-33 in human endothelial cells. (**a**–**g**) Relative mRNA levels in cells either not treated or treated with different concentrations of IL-33 (200 ng/ml, 1 μg/ml) during 6 h or 12 h were determined by qPCR. Primer sets included several genes (*SELE, NFkB2, ICAM1, VCAM1, IL-8*) that correspond to modulated proteins identified by the proteomic approach. Relative mRNA levels were calculated by normalizing the signals to those of actin. Results are shown as means with s.d. from 3 separate datapoints. P-values < 0.05 were considered significant.

To corroborate the results obtained using the global proteomic approach, we next performed qPCR experiments. These analyses revealed that expression of several proteins highly induced by IL-33 (SELE/E-selectin, ICAM1, VCAM1, IL-8 and p100/NFkB2), was upregulated at the mRNA level (Fig. 3). Induction of these factors was observed using two different concentrations of IL-33 (200 ng/ml and 1 μg/ml) and two different stimulation time points (6 h and 12 h). IL-6 mRNA was also induced after IL-33 treatment of primary human endothelial cells (Fig. 3), despite the fact we did not detect the protein in our proteomic experiments, maybe because the few peptides generated from tryptic cleavage of IL-6 were not efficiently ionized in the mass spectrometry experiments. Similar to IL-6, the ST2 receptor was not detected by proteomic analysis. Our qPCR experiments indicated that ST2 mRNA was upregulated in cells stimulated with IL-33 but the induction was not very strong (Fig. 3). The effects of the two concentrations of IL-33 were similar, except for the induction of IL-6 that was stronger after 12 h incubation with 1 μg/ml IL-33.

### Knockdown of endogenous nuclear IL-33 expression in primary human endothelial cells using two independent RNA silencing strategies

**Figure 4:**
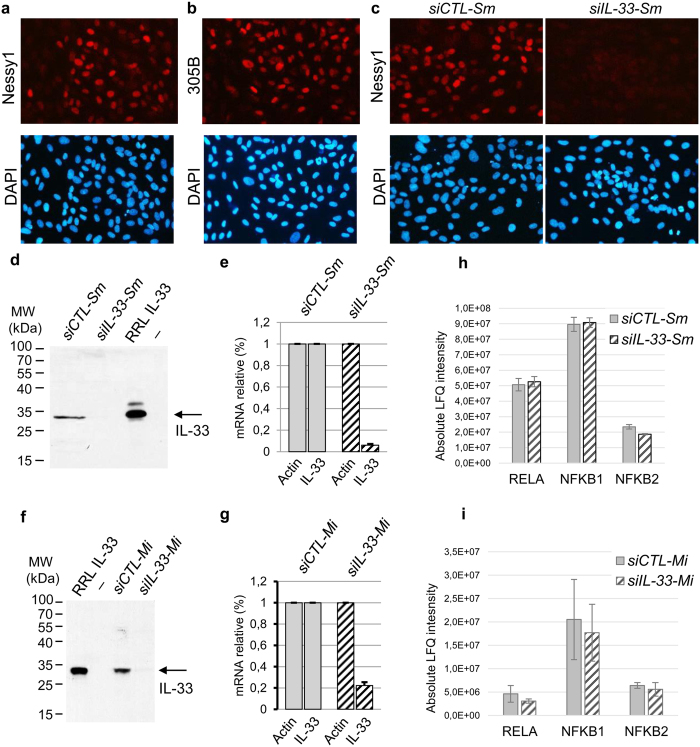
Knockdown of endogenous nuclear IL-33 expression in primary human endothelial cells using two independent RNA silencing strategies. (**a,b**) Nuclear expression of endogenous IL-33 in confluent monolayers of primary human endothelial cells. IL-33 protein (red) was detected by indirect immunofluorescence staining with anti-IL-33 mAbs Nessy1 (**a**) and 305B (**b**). Nuclei were counterstained with DAPI (blue). (**c**) Knockdown of endogenous nuclear IL-33 expression. IL-33 expression was analyzed by indirect immunofluorescence with Nessy1 mAb 48 h after transfection of a pool of *IL-33 siRNAs (siIL-33-Sm*) or control siRNA (*siCTL-Sm*). (**d–g**) Validation of the two independent RNA silencing strategies. Expression of IL-33 was analyzed at the protein level by western blot (**d,f**) and RNA level by qPCR (**e,g**), 72 h after the second siRNA transfection. Cells were treated with distinct pools of *IL-33 siRNAs*, *siIL-33-Sm* (**d,e**) or *siIL-33-Mi* (**f,g**), and corresponding controls, *siCTL-Sm* (**d,e**) and *siCTL-Mi* (**f,g**). RRL IL-33, human IL-33 protein produced by *in vitro* translation in rabbit reticulocyte lysates (**d,f**). For the qPCR experiments, relative mRNA levels were calculated by normalizing the signals to those of actin. Results are shown as means with s.d. from three separate datapoints. (**h,i**). Knockdown of endogenous nuclear IL-33 does not affect NFkB protein expression. Normalized protein quantities deduced from all peptides intensity values (LFQ intensities) are shown for NFkB p65 (RELA), NFkB p105 (NFKB1) and NFkB p100 (NFKB2). Endothelial cells were treated with *siIL-33-Sm* (**h**) or *siIL-33-Mi* (**i**), and corresponding controls. Results are shown as means with s.d. from three biological replicate experiments.

We then decided to apply the global proteomic approach to analyse the potential role of nuclear IL-33 in the regulation of endothelial cell protein expression. In order to obtain reliable experimental results, we used two independent RNA silencing strategies to knockdown endogenous IL-33 expression in endothelial cells (Fig. 4). We first confirmed the strong expression of endogenous nuclear IL-33 in confluent monolayers of primary human endothelial cells, using two independent anti-IL-33 antibodies validated in our previous studies^21^,^26^, Nessy-1 (Fig. 4a) and 305B (Fig. 4b). In agreement with previous observations^22^, confluency of the endothelial cells was critical for IL-33 expression. In the first knockdown strategy (RNA silencing strategy 1), we used a pool of four distinct siRNAs (Dharmacon *ON-TARGETplus SMARTpool IL-33 siRNAs*) that have been specifically modified for efficient silencing of the target gene with reduced off-target effects. This pool of *IL-33* siRNAs (*siIL-33-Sm*) abrogated expression of endogenous nuclear IL-33 as revealed by immunofluorescence staining (Fig. 4c, Nessy-1). Western blot (Fig. 4d, 305B) and qPCR (Fig. 4e) analyses confirmed the efficient knockdown of endogenous IL-33 expression. Human IL-33 protein produced by *in vitro* translation in rabbit reticulocyte lysates (RRL IL-33) was used as a control for the western blot. The second knockdown strategy (RNA silencing strategy 2) was based on the use of an independent pool of three siRNAs targeting *IL-33*, predesigned by another provider using new and critical siRNA design rules (Sigma *MISSION Predesigned Il-33 siRNAs* based on Rosetta siRNA design algorithm). Similar to the first pool, this second pool of *IL-33* siRNAs (*siIL-33-Mi*) efficiently reduced endogenous IL-33 protein (Fig. 4f, 305B) and mRNA (Fig. 4g) expression in primary human endothelial cells.

### Global proteomic analyses indicate that knockdown of endogenous nuclear IL-33 had no reproducible effect on the endothelial cell proteome

**Figure 5:**
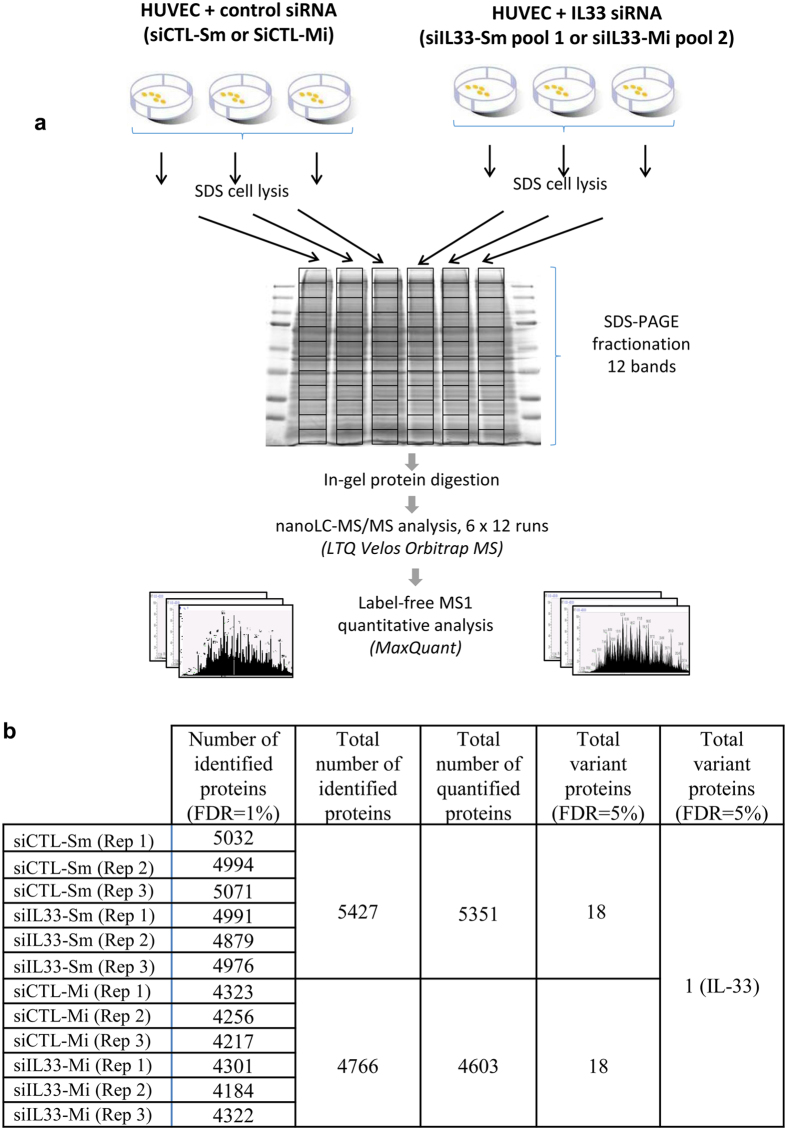
High throughput mass spectrometry analysis of the endothelial cell proteome after silencing of endogenous nuclear IL-33 expression. (**a**) Experimental design. Three independent biological experiments were performed for each RNA silencing strategy. Primary human endothelial cells (HUVECs) were treated with control or *IL-33* siRNAs (*siIL-33-Sm* pool, RNA silencing strategy 1; *siIL-33-Mi* pool, RNA silencing strategy 2). Total cell lysates prepared from confluent monolayers 72 h after the second siRNA transfection were fractionated on six parallel gel lanes, cut into 12 gel bands, in-gel digested, and the corresponding peptide digests were analyzed by nanoLC-MS/MS. (**b**) The table indicates the number of proteins identified for each condition, and the total number of proteins identified and quantified in each experiment, as well as the number of variant proteins detected with each RNA silencing strategy.

We next compared endothelial cells treated with IL-33 siRNA pools or control siRNAs on a global scale using a high-throughput proteomic approach (Fig. 5a) similar to the one developed to analyse the effect of extracellular IL-33 (Fig. 1a). Three biological replicates were performed for each RNA silencing strategy, and more than 4500 distinct proteins were quantified in both cases (5351 and 4603 proteins quantified for RNA silencing strategy 1 and 2, respectively) (Fig. 5b). A permutation-based false discovery rate of 5% was applied to identify modulated proteins. Since the two independent RNA silencing strategies induced similar downregulation of endogenous IL-33 (Fig. 4d,f), we reasoned that any protein modulated by nuclear IL-33 should be identified using the two knockdown strategies. Strikingly, only one modulated protein was reproducibly identified using the two independent RNA silencing strategies and it was IL-33 itself (Fig. 5b and Supplementary Table 2). The identification of endogenous IL-33 as a downregulated protein provided additional evidence that our proteome-wide analyses are able to pick up modulated proteins in endothelial cells. Thus, our global proteomic analyses and carefully controlled knockdown experiments demonstrated that silencing of *IL-33* mRNA in primary human endothelial cells efficiently reduced endogenous IL-33 protein expression but had no other reproducible effect on the endothelial cell proteome.

Nuclear IL-33 has previously been proposed to function as a direct transcriptional activator of *NFkB* that regulates NFkB p65 basal expression in endothelial cells^43^. However, these studies were performed using a single siRNA (not a pool) and the results are likely to be explained by off target effects of the siRNA. Indeed, in our knockdown experiments using two independent RNA silencing strategies (based on siRNA pools), we did not find any evidence for a role of nuclear IL-33 in the regulation of NFkB p65 (RELA), NFkB p105 (NFKB1) and NFkB p100 (NFKB2) protein expression in endothelial cells (Fig. 4h,i).

## Discussion

In this manuscript, we addressed an important question in IL-33 biology, the relative roles of extracellular IL-33 cytokine and endogenous intracellular nuclear IL-33 in the regulation of protein expression. Using a global proteomic approach in primary human endothelial cells, and two independent RNA silencing strategies, we did not find any evidence for a role of endogenous nuclear IL-33 in the modulation of the endothelial cell proteome. In contrast, extracellular IL-33 was found to induce the expression of many proteins associated with inflammatory responses in endothelial cells. Together, these proteome-wide analyses do not support the previous view of IL-33 as a dual function protein, acting both as a nuclear transcription factor and an extracellular cytokine^2^.

Endothelial cells constituted an ideal system for our studies because they represent a major source of endogenous IL-33 in human tissues^2^,^21^,^22^, and they express high levels of the cytokine in their nuclei both *in vivo*^2^,^21^,^22^,^44^ and *ex vivo*^22^,^26^. In addition, endothelial cells express the ST2 receptor and can respond to extracellular IL-33 cytokine^37^,^38^. Based on the observation that IL-33 exhibits transcriptional repressor properties in Gal4-reporter assays^2^,^32^, we initially proposed that IL-33 may function both as an extracellular cytokine and as an intracellular nuclear factor with transcriptional regulatory properties^2^,^32^. Others suggested that the transcriptional repressor function of IL-33 may be involved in the control of endothelial cell activation^22^,^33^. Our present analyses on a global scale indicate that endogenous nuclear IL-33 does not regulate endothelial cell protein expression. These results were totally unexpected because nuclear localization of IL-33 is an evolutionary conserved fundamental property of the protein that has been observed in all producing cells both in human and mouse tissues^21^,^22^,^23^. However, this lack of nuclear function of IL-33 in human endothelial cells at baseline is consistent with our previous observation that IL-33 is generally not constitutively expressed in endothelial cells from blood vessels in mouse tissues^23^. It will be important to confirm our observations made in endothelial cells, in other cell types producing IL-33, particularly mucosal epithelial cells, and during chronic active disease where nuclear expression of IL-33 has been shown to be significantly increased.

Our findings suggest that nuclear localization and chromatin-association of IL-33 have been selected during evolution for purposes different from regulation of gene expression. Interestingly, it has recently been shown in a gene-targeted knock-in mouse model, that deletion of the chromatin-binding nuclear domain of IL-33 results in constitutive extracellular release of the cytokine in serum, multi-organ inflammation and death of the mice after 4 months^28^. Importantly, the profound inflammatory phenotype observed was not due to the loss of a nuclear function of IL-33, but rather to the extracellular cytokine activity of IL-33, since it was completely prevented in *ST2*-deficient mice^28^. The main purpose of IL-33 nuclear localization may thus be the regulation of its extracellular cytokine activity, rather than regulation of gene expression. This is a critical role since it is essential for survival of the organism^28^. It may explain why the N-terminal nuclear domain of IL-33 exhibits a high degree of evolutionary conservation, like the C-terminal IL-1-like domain^2^,^32^. The lack of a nuclear function of IL-33 in regulation of gene (or protein) expression would be also consistent with the observation that unchallenged IL-33 deficient mice are healthy^23^,^45^, and have no obvious phenotypes in organs known to contain high numbers of IL-33 producing cells (i.e. lungs and lymphoid organs). Regulation of IL-33 cytokine activity by nuclear compartmentalization (nuclear sequestration)^28^ and inactivation by caspases^26^,^27^ are likely to be related to the proposed mode of action of IL-33 as an alarm signal (alarmin or endogenous danger signal)^3^. These modes of regulation may have been selected because IL-33 protein is constitutively expressed at high levels in healthy mice and humans^21^,^22^,^23^ and it is thus necessary to limit its potent pro-inflammatory activities in the absence of danger, in order to prevent aberrant and lethal inflammation^3^,^28^.

IL-33 is constitutively expressed in tissues during homeostasis^21^,^23^, but its expression can be further increased during inflammation^3^,^4^,^23^,^25^. Upon tissue damage and cell death (or cellular stress), for instance following infection with viruses or parasites, IL-33 can be released from producing-cells and act as a potent pro-inflammatory cytokine^3^,^4^. Our global proteomic analyses in primary human endothelial cells indicated that extracellular IL-33 cytokine induces the expression of many proteins involved in inflammatory responses and associated with the NF-kB pathway (Fig. 2). The effect of IL-33 on endothelial cell proteome was similar to those of IL-1β and TNFα/INFγ^40^. Several proteins found in our high-throughput proteomic analyses, including IL-8, E-selectin, ICAM1 and VCAM1, have previously been identified using flow cytometry and/or ELISA assays^37^,^38^,^46^. Most others proteins identified in our differential analysis have not previously been described to be upregulated by IL-33 in human endothelial cells, although many of them have been shown to be induced at the mRNA level in a microarray study^38^. These later proteins included cytokines/chemokines (CXCL6, CCL20, IL-27b), cell surface proteins (ICOS ligand, syndecan-4, membrane transporters) and proteins involved in antigen processing and presentation through MHC class I molecules (immunoproteasome subunits, TAP1, TAP2, TABP, CTSS and HLA molecules). Finally, IL-33 has recently been shown to upregulate expression of GM-CSF, M-CSF^47^ and tissue factor^48^ in human endothelial cells, but these proteins were not identified in our global proteomic analyses.

In the present study, we have not determined whether endogenous nuclear IL-33 modulates the response to extracellular IL-33 or other pro-inflammatory cytokines, but a previous report analyzing induction of E-selectin, one of the major NF-kB targets modulated by IL-33 in endothelial cells (Fig. 2), showed that modification of nuclear IL-33 does not alter the sensitivity to extracellular IL-33^38^. Therefore, although overexpressed IL-33 has been shown to dampen NF-kB stimulated gene transcription in transfected cells^33^, endogenous nuclear IL-33 does not appear to play such a role^38^. It has recently been reported that overexpression of nuclear IL-33 *in vivo* in CMV-IL-33 transgenic mice results in a severe inflammatory phenotype associated with growth retardation and paw swelling^49^. Interestingly, this neutrophil-dominated inflammatory phenotype was completely suppressed in *ST2*-deficient mice^49^ indicating that it is due to the activity of extracellular IL-33 cytokine but not to the effect of IL-33 in the nucleus. Therefore, although we can’t formally exclude a role for IL-33 in the nucleus during inflammatory processes, particularly when it is upregulated, we consider this possibility unlikely.

In conclusion, our global proteomic analyses indicate that extracellular, but not nuclear, IL-33 regulates protein expression in human endothelial cells. Therefore, they do not support the view of IL-33 as a dual function protein with roles in the regulation of gene expression in the nucleus. Instead, they suggest, together with recent observations in mice^28^, that the main purpose of IL-33 nuclear localization and chromatin association is the regulation of its potent extracellular cytokine activity. Nuclear compartmentalization (or sequestration) of IL-33 may thus have been selected during evolution as a mean to maintain immune homeostasis and protect the organism from lethal inflammation.

## Methods

### Stimulation of endothelial cells with IL-33 cytokine

Primary human umbilical vein endothelial cells (HUVECs, Praxcell) were grown on gelatin-coated (0.2% gelatin, Sigma) Petri dishes in endothelial cell growth medium (ECGM, Promocell), supplemented with 20% fetal calf serum and 100 μg/ml heparin (Sigma). Treatment with IL-33 cytokine was performed by incubating the endothelial cells for 6 h (proteomics and qPCR), 12 h (qPCR) or 24 h (proteomics) with 100 ng/ml (proteomics), 200 ng/ml (qPCR) or 1 μg/ml (qPCR) recombinant human IL-33 mature form IL-33_95–270_^29^,^30^ in ECGM medium supplemented with 20% fetal calf serum and heparin (100 μg/ml).

### Silencing of endogenous IL-33 in endothelial cells

Knockdown of IL-33 expression in primary human umbilical vein endothelial cells (HUVECs) was performed using two independent strategies. ON-TARGET plus SMARTpool IL-33 siRNA duplexes (Dharmacon; IL-33 siRNAs J-015122-09, J-015122-10, J-015122-11, J-015122-12), and MISSION Predesigned IL-33 siRNAs (Sigma; SASI_Hs01_00129936, SASI_Hs01_00129937, SASI_Hs01_00129940), were used in RNA silencing strategies 1 and 2, respectively, together with their corresponding controls (Dharmacon *ON-TARGET plus* SiControl; Sigma MISSION SiControl SiC001). Two successive transfections of endothelial cell monolayers were performed at 24 h interval by incubating cells (300 000 cells/well in 6-well plates) for 6 hours with siRNA duplexes at 50 nM final concentrations in Oligofectamine and serum-free Opti-MEM-1 (Invitrogen). siRNA duplexes (10 μl of stock solution at 10 μM) and oligofectamine (10 μl) were mixed in 0.4 ml Opti-MEM-1 during 20 min at room temperature before addition to the cells in Opti-MEM-1 medium (2 ml final volume). After the 6 h incubation with siRNA/oligofectamine mix, cells were washed and 3 ml complete ECGM medium was added to each well. After the second siRNA transfection, the cells were transferred to 10 cm gelatin-coated Petri dishes (3 × 10^6 ^cells/10 ml) and grown in complete ECGM medium (20% fetal calf serum, 100 μg/ml heparin) for 24 h-72 h. Media was replaced every day to eliminate cells that detached from the plates. Confluent monolayers were analyzed by qPCR, western blot, immunofluorescence, and high-throughput proteomics.

### Quantitative PCR (qPCR)

Total RNA was isolated using the Absolute RNA Kit from Stratagene (Agilent Technologies) and cDNAs were synthesized using SuperSript III First strand cDNA synthesis system for RT-PCR (Invitrogen) according to manufacturer’s instructions. qPCR was performed using the ABI7500 Prism SDS Real-Time PCR Detection System (Applied Biosystems) with a SYBR Green PCR Master Mix kit (Applied Biosystems) and a standard temperature protocol. The results are expressed as relative quantities and calculated using the 2-ΔΔCT method. Three separate qPCR experiments were performed. Actin was used as a control gene for normalization. Primers were purchased from Qiagen (QuantiTect primer assay, *IL-33, IL-8, IL-6, SELE*) or Sigma Genosys (*Actin, NFKB2, ICAM1, VCAM1, ST2*).

### Western blot

Proteins were fractionated by SDS-PAGE, electroblotted and detected with mAbs to human IL-33, Nessy-1 (1/1000, Alexis Biochemicals) or 305B (1/1000, Alexis Biochemicals), followed by HRP-conjugated goat anti-mouse polyclonal antibodies (1/10000; Promega), and finally an enhanced chemiluminescence kit (GE Healthcare). *In vitro* translated full length human IL-33_1–270_^26^ was used as a positive control for the western blot.

### Immunofluorescence microscopy

Cells were fixed for 15 min at room temperature in PBS containing 3.7% formaldehyde, and permeabilized 5 min at room temperature in PBS containing 0.1% Triton X-100. Permeabilized cells were then blocked with PBS, 1% bovine serum albumin, and incubated 2 h at room temperature with mAbs to human IL-33 (Nessy-1 or 305B, 1/400). Cells were then washed three times and incubated for 1 h with Cy3-conjugated goat anti-mouse secondary antibodies (Amersham, 1/1000). After extensive washing in PBS and counterstaining with DAPI, samples were air-dried and mounted in Mowiol. Images were collected on a Nikon Eclipse TE300 fluorescence microscope equipped with a Nikon digital camera DXM1200 (Nikon).

### Protein sample processing for MS

Cells were lysed in a buffer containing 2% of SDS and sonicated, and protein concentration was determined by detergent-compatible assay (DC assay, Biorad). Protein samples were reduced in Laemli buffer (final composition 25 mM DTT, 2% SDS, 10% glycerol, 40 mM Tris pH 6.8), 5 min at 95 °C. Cysteine residues were alkylated by addition of iodoacetamide at a final concentration of 90 mM and incubation during 30 min at room temperature in the dark. During the alkylation reaction, the pH of the samples was adjusted using small amounts of Tris 1 M, pH 8. Protein samples were loaded on a home-made 1D SDS-PAGE gel (separating gel 1.5 mm × 5 cm, 12% acrylamide polymerized in SDS 0.1%, Tris 375 mM pH 8.8 and stacking gel 1.5 mm × 1.5 cm, 4% acrylamide polymerized in SDS 0.1%, Tris 125 mM. Electrophoretic migration was performed in order to fractionate each protein sample (100 μg) into 12 gel bands. For replicate and comparative analyses, samples were processed on adjacent migration lanes that were cut simultaneously with a long razor blade. Gel slices were washed by two cycles of incubation in 100 mM ammonium bicarbonate, 15 min, 37 °C, followed by 100 mM ammonium bicarbonate/acetonitrile (1:1), 15 min, 37 °C. Proteins were digested by 0.6 μg of modified sequencing grade trypsin (Promega) in 50 mM ammonium bicarbonate, overnight at 37 °C. The resulting peptides were extracted from the gel by incubation in 50 mM ammonium bicarbonate, 15 min, 37 °C and twice in 10% formic acid/acetonitrile (1:1), 15 min, 37 °C. The three collected extractions were pooled with the initial digestion supernatant, dried in speed-vac and resuspended with 17 μL of 5% acetonitrile, 0.05% trifluoroacetic acid (TFA).

### NanoLC-MS/MS analysis

Resulting peptides were analyzed by nanoLC-MS/MS using an Ultimate3000 system (Dionex, Amsterdam, The Netherlands) coupled to an LTQ-Orbitrap Velos mass spectrometer (Thermo Fisher Scientific, Bremen, Germany). Five μL of each sample were loaded on a C-18 precolumn (300 μm ID × 5 mm, Dionex) at 20 μL/min in 5% acetonitrile, 0.05% TFA. After 5 minutes desalting, the precolumn was switched online with the analytical C-18 column (75 μm ID × 15 cm, PepMap C18, Dionex) equilibrated in 95% solvent A (5% acetonitrile, 0.2% formic acid) and 5% solvent B (80% acetonitrile, 0.2% formic acid). Peptides were eluted using a 5 to 50% gradient of solvent B during 80 min at 300 nL/min flow rate. The LTQ-Orbitrap Velos was operated in data-dependent acquisition mode with the XCalibur software. Survey scan MS were acquired in the Orbitrap on the 300–2000 m/z range with the resolution set to a value of 60000. The 10 most intense ions per survey scan were selected for CID fragmentation and the resulting fragments were analyzed in the linear trap (LTQ). Dynamic exclusion was employed within 60 seconds to prevent repetitive selection of the same peptide.

The study of the effect of stimulation with extracellular IL-33 leaded to the acquisitions of 108 raw MS files, corresponding to the analysis of 12 gel fractions for each of the 3 conditions (no stimulation; IL-33-6h; IL-33-24h), and the corresponding dataset has been deposited to the ProteomeXchange repository^50^ with the identifier PXD004117. Each of the two different endogenous IL-33 silencing studies yielded 72 raw MS files corresponding to 12 gel fractions for each of the 2 conditions (Ctrl; siRNA). The total dataset corresponding to siRNA experiments has been deposited to ProteomeXchange with the identifier PXD004141.

### Database search and quantitative analysis

Raw mass spectrometry files were processed with the MaxQuant software (version 1.5.2.8) for database search with the Andromeda search engine and for quantitative analysis (Individual processing for each of the 3 MS datasets described above). Data were searched against Human entries of the Swissprot protein database (UniProtKB/Swiss-Prot Knowledgebase release 2016/01, Human taxonomy, 20194 entries). Carbamidomethylation of cysteines was set as a fixed modification whereas oxidation of methionine and protein N-terminal acetylation were set as variable modifications. Specificity of trypsin digestion was set for cleavage after K or R, and two missed trypsin cleavage sites were allowed. The precursor mass tolerance was set to 20 ppm for the first search and 4.5 ppm for the main Andromeda database search. The mass tolerance in MS/MS mode was set to 0.8 Da. Minimum peptide length was set to 7 amino acids, and minimum number of unique peptides was set to 1. Andromeda results were validated by the target-decoy approach using a reverse database at both a peptide and protein FDR of 1%. For label-free relative quantification of the samples, the “match between runs” option of MaxQuant was enabled with a time window of 3 min, to allow cross-assignment of MS features detected in the different runs.

To perform relative quantification between proteins identified in different biological conditions, we used the LFQ metric from the MaxQuant “protein group.txt” output (reflecting a normalized protein quantity deduced from all peptides intensity values). For each comparison, only proteins which were quantified in the 3 replicate experiments (3 LFQ values retrieved by MaxQuant) in at least one of the biological conditions were considered for further processing and statistical analysis. Remaining missing values for the other biological condition were considered to be associated to non-expressed or undetectable proteins when the LFQ was not extracted in at least 2 out of the 3 MS replicates, and were then replaced by a constant noise value determined independently for each analytical run as the 1% percentile of the total protein population. LFQ values were processed for statistical analysis with the Perseus software (version 1.5.3.0) using two-sided Student t-test and permutation-based FDR correction for multiple hypothesis testing. S0 constant for variance correction was set at 0.2 for all comparisons, and the wanted level of FDR was set at 5% to select variant proteins.

## Additional Information

**How to cite this article**: Gautier, V. *et al.* Extracellular IL-33 cytokine, but not endogenous nuclear IL-33, regulates protein expression in endothelial cells. *Sci. Rep.* **6**, 34255; doi: 10.1038/srep34255 (2016).

## Supplementary Material

Supplementary Information

Supplementary Table 1

Supplementary Table 2
