# Supplementary material for: Extracellular IL-33 cytokine, but not endogenous nuclear IL-33, regulates protein expression in endothelial cells
Source: Sci Rep. 2016 Oct 3;6:34255. doi: 10.1038/srep34255 (PMC5046127; doi:10.1038/srep34255)
Supplement: Supplementary Information [file srep34255-s1.pdf]

Supplementary Information

# Extracellular IL-33 cytokine, but not endogenous nuclear IL-33, regulates protein expression in endothelial cells

**Violette Gautier[1], Corinne Cayrol[1], Dorian Farache[1], Stéphane Roga[1], Bernard Monsarrat[1], Odile Burlet-Schiltz[1], Anne Gonzalez de Peredo[1] and Jean-Philippe Girard[1,*]**

[1]Institut de Pharmacologie et de Biologie Structurale, Université de Toulouse, CNRS, UPS, Toulouse, France

**Supplementary Figure 1:** Multi scatter plot of MS data showing repeatability between replicate experiments (denoted A, B and C for each condition) and global proteome stability between biological conditions (Control, Il33 stimulation 6h, Il33 stimulation 24h). All LFQ intensity values for each protein were plotted after log2 transformation. Pearson correlation coefficients are indicated for each pairwise comparison.

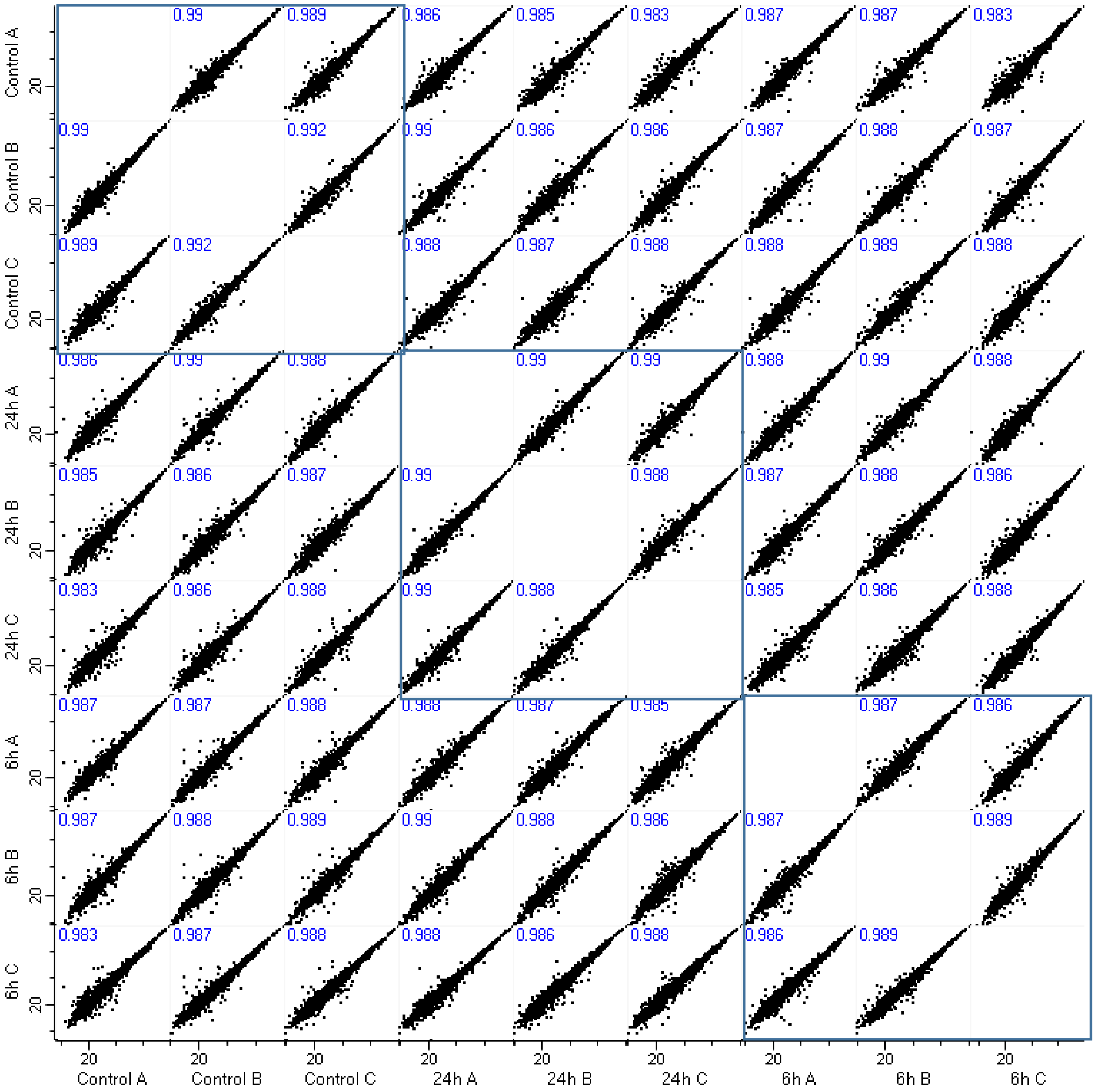


| Name | Contro | Contro | Contro | 24h A | 24h B | 24h C | 6h A | 6h B | 6h C |
|---|---|---|---|---|---|---|---|---|---|
| Control A | 1 | 0.99 | 0.989 | 0.986 | 0.985 | 0.983 | 0.987 | 0.987 | 0.983 |
| Control B | 0.99 | 1 | 0.992 | 0.99 | 0.986 | 0.986 | 0.987 | 0.988 | 0.987 |
| Control C | 0.989 | 0.992 | 1 | 0.988 | 0.987 | 0.988 | 0.988 | 0.989 | 0.988 |
| 24h A | 0.986 | 0.99 | 0.988 | 1 | 0.99 | 0.99 | 0.988 | 0.99 | 0.988 |
| 24h B | 0.985 | 0.986 | 0.987 | 0.99 | 1 | 0.988 | 0.987 | 0.988 | 0.986 |
| 24h C | 0.983 | 0.986 | 0.988 | 0.99 | 0.988 | 1 | 0.985 | 0.986 | 0.988 |
| 6h A | 0.987 | 0.987 | 0.988 | 0.988 | 0.987 | 0.985 | 1 | 0.987 | 0.986 |
| 6h B | 0.987 | 0.988 | 0.989 | 0.99 | 0.988 | 0.986 | 0.987 | 1 | 0.989 |
| 6h C | 0.983 | 0.987 | 0.988 | 0.988 | 0.986 | 0.988 | 0.986 | 0.989 | 1 |

Pearson coefficients for pairwise comparisons

**Supplementary Figure 2:** Box-plots of MS intensity data (LFQ intensity values after log2 transformation) obtained from replicate experiments, in the 3 biological conditions (Control, Il33 stimulation 6h, Il33 stimulation 24h), for all proteins showing significant expression changes in either 24h versus control or 6h versus control comparisons.

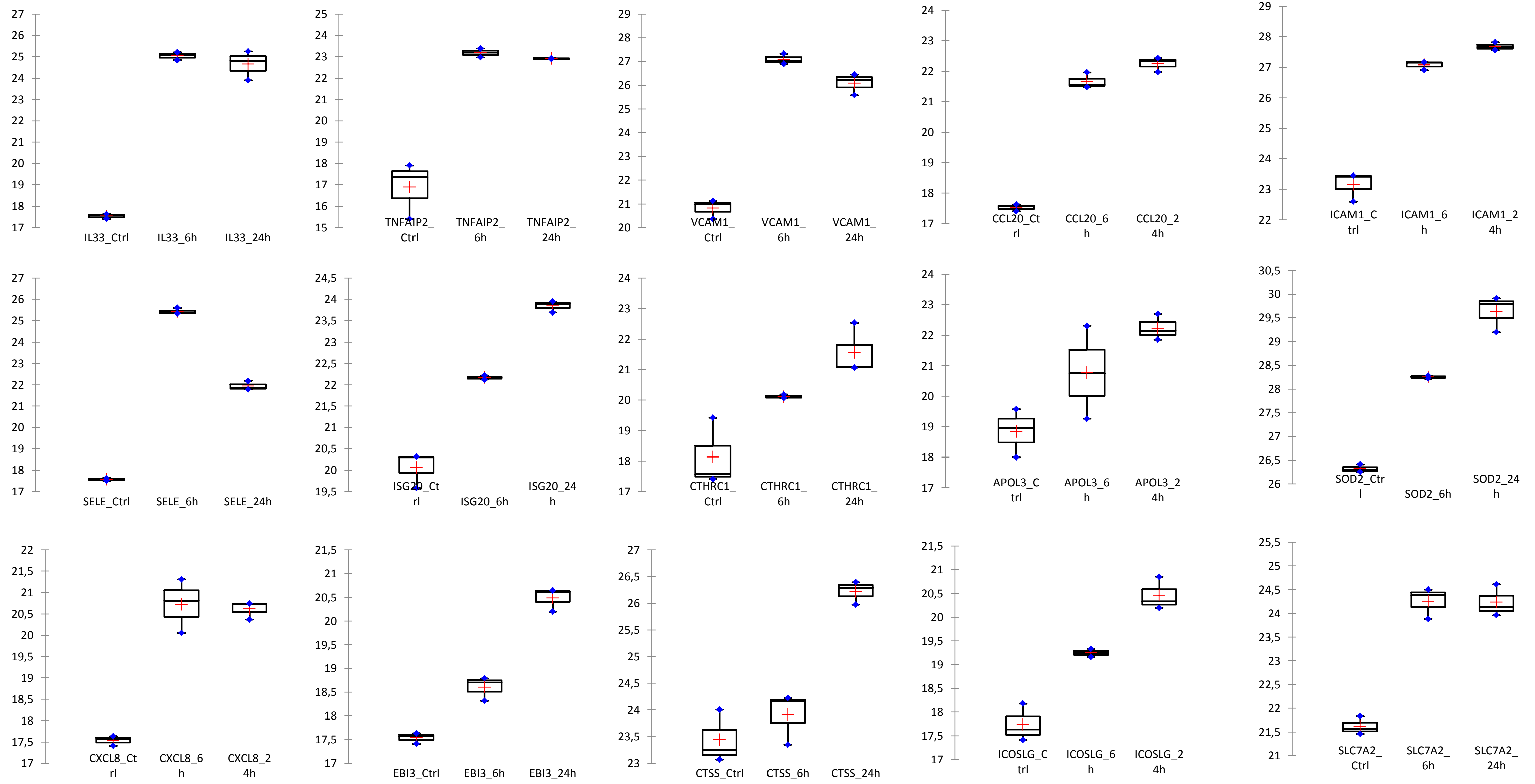

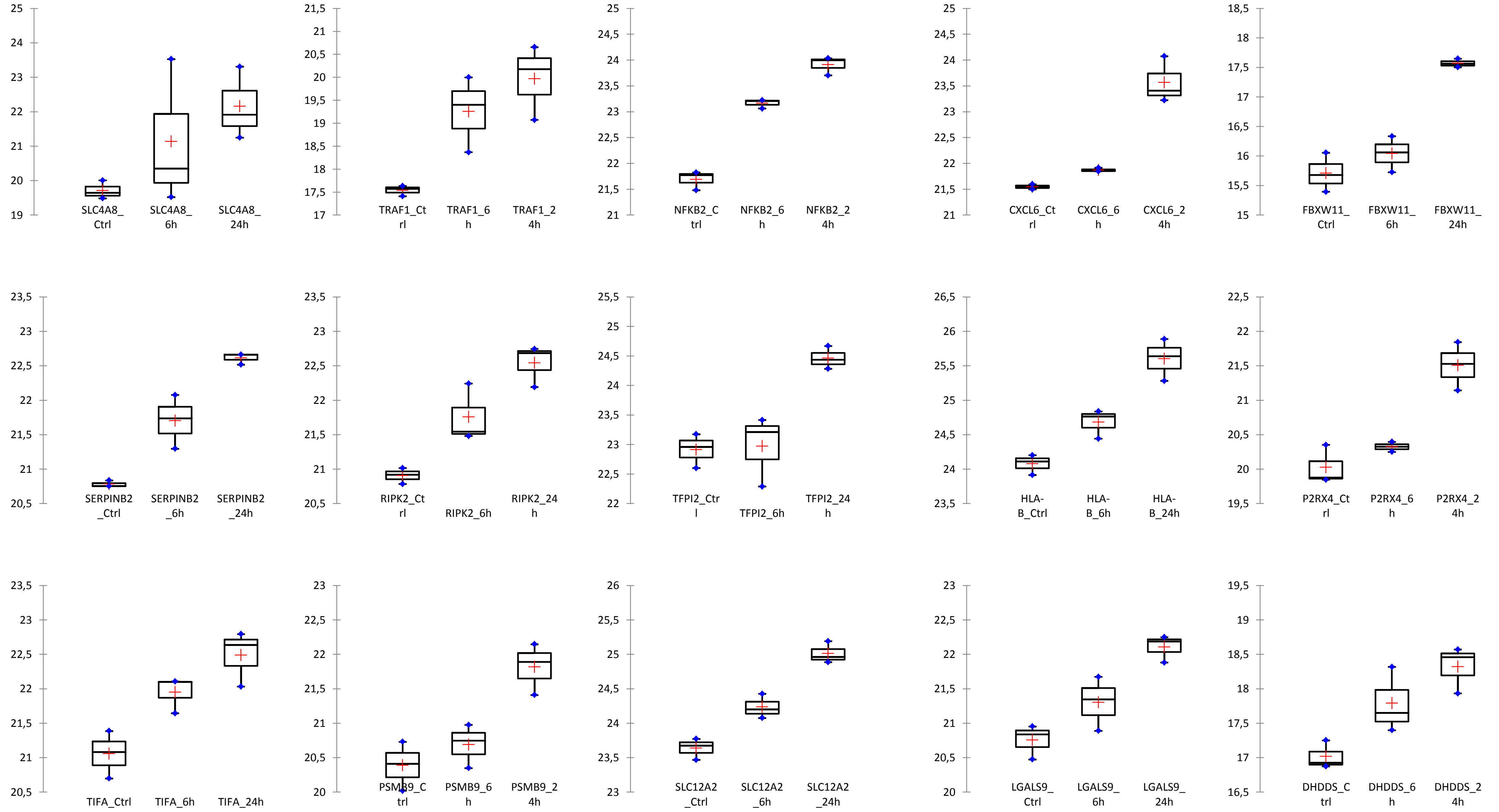
SLC4A8_ Ctrl
SLC4A8_ 6h
SLC4A8_ 24h
TRAF1_Ct rl
TRAF1_6 h
TRAF1_2 4h
NFKB2_C trl
NFKB2_6 h
NFKB2_2 4h
CXCL6_Ct rl
CXCL6_6 h
CXCL6_2 4h
FBXW11_ Ctrl
FBXW11_ 6h
FBXW11_ 24h
SERPINB2 _Ctrl
SERPINB2 _6h
SERPINB2 _24h
RIPK2_Ct rl
RIPK2_6h
RIPK2_24 h
TFPI2_Ctr l
TFPI2_6h
TFPI2_24 h
HLA- B_Ctrl
HLA- B_6h
HLA- B_24h
P2RX4_Ct rl
P2RX4_6 h
P2RX4_2 4h
TIFA_Ctrl
TIFA_6h
TIFA_24h
PSMB9_C trl
PSMB9_6 h
PSMB9_2 4h
SLC12A2 _Ctrl
SLC12A2 _6h
SLC12A2 _24h
LGALS9_ Ctrl
LGALS9_ 6h
LGALS9_ 24h
DHDDS_C trl
DHDDS_6 h
DHDDS_2 4h

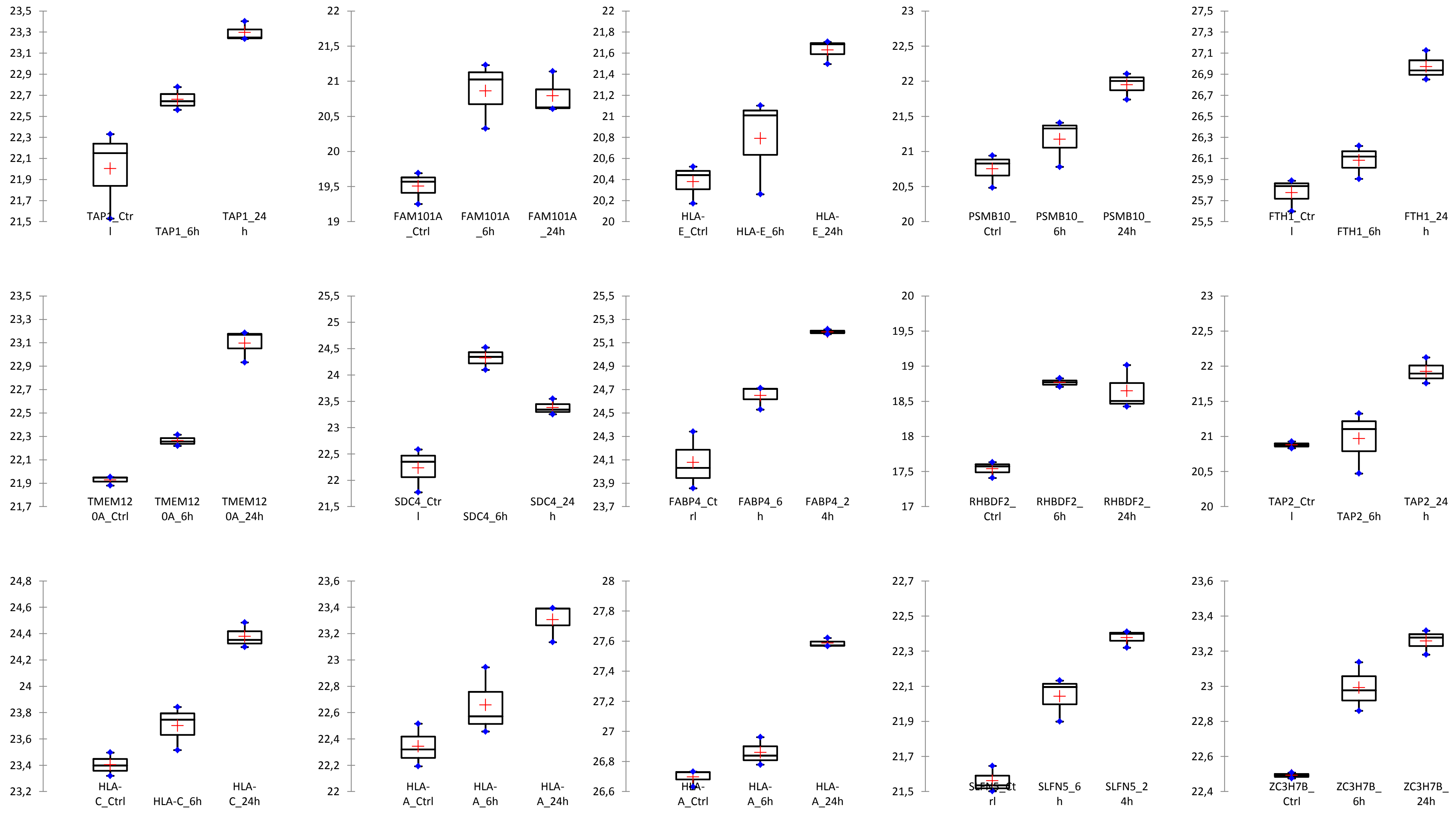

23,5
23,3
23,1
22,9
22,7
22,5
22,3
22,1
21,9
21,7
21,5
TAP1_Ctrl
TAP1_6h
TAP1_24h
22
21,5
21
20,5
20
19,5
19
FAM101A_Ctrl
FAM101A_6h
FAM101A_24h
22
21,8
21,6
21,4
21,2
21
20,8
20,6
20,4
20,2
20
HLA-E_Ctrl
HLA-E_6h
HLA-E_24h
23
22,5
22
21,5
21
20,5
20
PSMB10_Ctrl
PSMB10_6h
PSMB10_24h
27,5
27,3
27,1
26,9
26,7
26,5
26,3
26,1
25,9
25,7
25,5
FTH1_Ctrl
FTH1_6h
FTH1_24h
23,5
23,3
23,1
22,9
22,7
22,5
22,3
22,1
21,9
21,7
TMEM120A_Ctrl
TMEM120A_6h
TMEM120A_24h
25,5
25
24,5
24
23,5
23
22,5
22
21,5
SDC4_Ctrl
SDC4_6h
SDC4_24h
25,5
25,3
25,1
24,9
24,7
24,5
24,3
24,1
23,9
23,7
FABP4_Ctrl
FABP4_6h
FABP4_24h
20
19,5
19
18,5
18
17,5
17
RHBDF2_Ctrl
RHBDF2_6h
RHBDF2_24h
23
22,5
22
21,5
21
20,5
20
TAP2_Ctrl
TAP2_6h
TAP2_24h
24,8
24,6
24,4
24,2
24
23,8
23,6
23,4
23,2
HLA-C_Ctrl
HLA-C_6h
HLA-C_24h
23,6
23,4
23,2
23
22,8
22,6
22,4
22,2
22
HLA-A_Ctrl
HLA-A_6h
HLA-A_24h
28
27,8
27,6
27,4
27,2
27
26,8
26,6
HLA-A_Ctrl
HLA-A_6h
HLA-A_24h
22,7
22,5
22,3
22,1
21,9
21,7
21,5
SLFN5_Ctrl
SLFN5_6h
SLFN5_24h
23,6
23,4
23,2
23
22,8
22,6
22,4
ZC3H7B_Ctrl
ZC3H7B_6h
ZC3H7B_24h

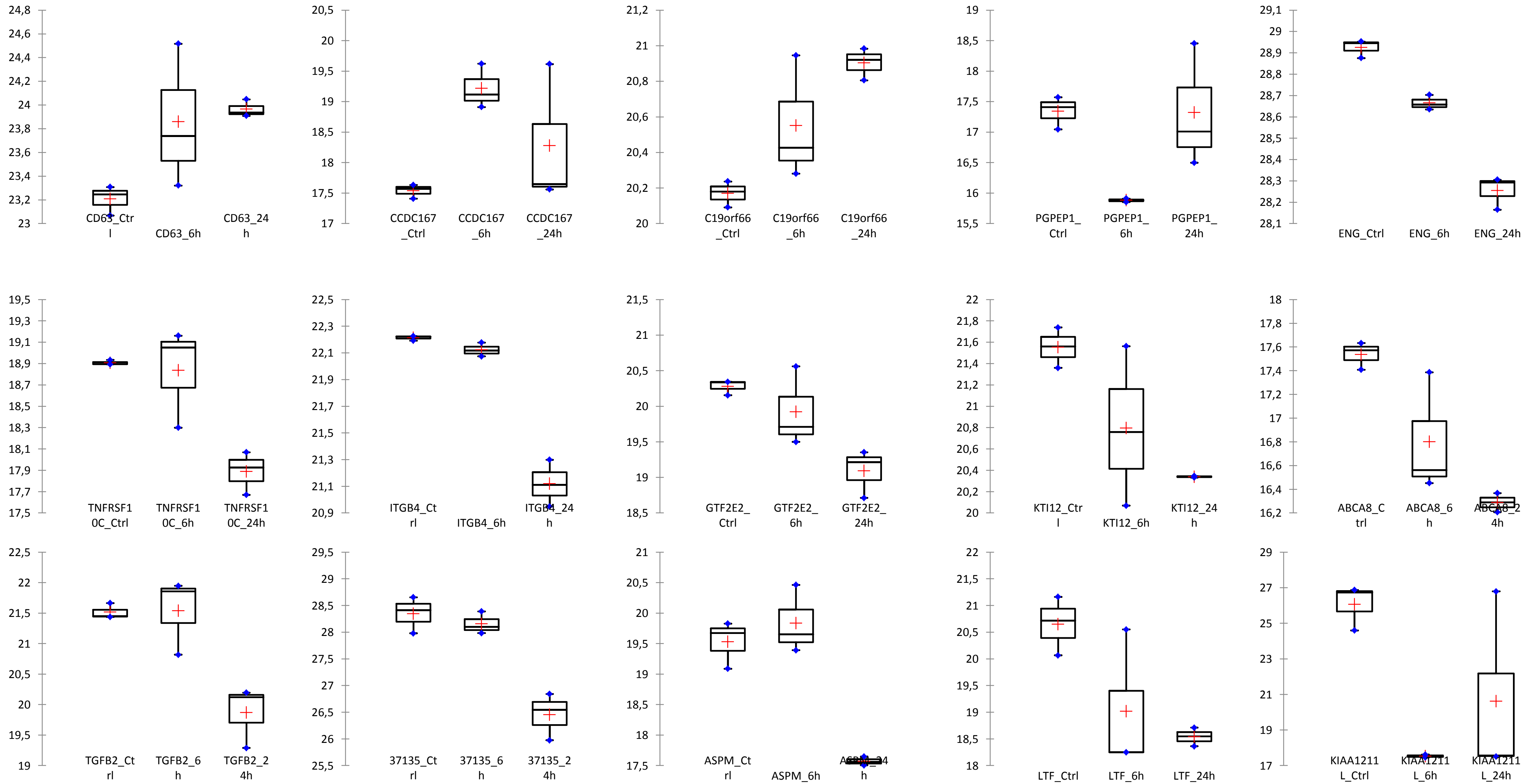
24,8 24,6 24,4 24,2 24 23,8 23,6 23,4 23,2 23
CD63_Ctrl CD63_6h CD63_24h
20,5 20 19,5 19 18,5 18 17,5 17
CCDC167_Ctrl CCDC167_6h CCDC167_24h
21,2 21 20,8 20,6 20,4 20,2 20
C19orf66_Ctrl C19orf66_6h C19orf66_24h
19 18,5 18 17,5 17 16,5 16 15,5
PGPEP1_Ctrl PGPEP1_6h PGPEP1_24h
29,1 29 28,9 28,8 28,7 28,6 28,5 28,4 28,3 28,2 28,1
ENG_Ctrl ENG_6h ENG_24h
19,5 19,3 19,1 18,9 18,7 18,5 18,3 18,1 17,9 17,7 17,5
TNFRSF10C_Ctrl TNFRSF10C_6h TNFRSF10C_24h
22,5 22,3 22,1 21,9 21,7 21,5 21,3 21,1 20,9
ITGB4_Ctrl ITGB4_6h ITGB4_24h
21,5 21 20,5 20 19,5 19 18,5
GTF2E2_Ctrl GTF2E2_6h GTF2E2_24h
22 21,8 21,6 21,4 21,2 21 20,8 20,6 20,4 20,2 20
KTI12_Ctrl KTI12_6h KTI12_24h
18 17,8 17,6 17,4 17,2 17 16,8 16,6 16,4 16,2
ABCA8_Ctrl ABCA8_6h ABCA8_24h
22,5 22 21,5 21 20,5 20 19,5 19
TGFB2_Ctrl TGFB2_6h TGFB2_24h
29,5 29 28,5 28 27,5 27 26,5 26 25,5
37135_Ctrl 37135_6h 37135_24h
21 20,5 20 19,5 19 18,5 18 17,5
ASPM_Ctrl ASPM_6h ASPM_24h
22 21,5 21 20,5 20 19,5 19 18,5 18
LTF_Ctrl LTF_6h LTF_24h
29 27 25 23 21 19 17
KIAA1211L_Ctrl KIAA1211L_6h KIAA1211L_24h
